# Copper Deficiency Presenting With Bicytopenia During Long-Term Parenteral Nutrition for Short Bowel Syndrome: A Case Report and Literature Review

**DOI:** 10.7759/cureus.83688

**Published:** 2025-05-07

**Authors:** Marie Tachikawa, Dai Keino, Yu Kimizuka, Hiroaki Goto, Naomi Hatabu, Hidehito Usui, Norihiko Kitagawa, Masakatsu Yanagimachi

**Affiliations:** 1 Department of General Medicine/Division of Hematology/Oncology, Kanagawa Children's Medical Center, Yokohama, JPN; 2 Division of Hematology/Oncology, Kanagawa Children’s Medical Center, Yokohama, JPN; 3 Department of Endocrinology and Metabolism, Kanagawa Children’s Medical Center, Yokohama, JPN; 4 Department of Surgery, Kanagawa Children’s Medical Center, Yokohama, JPN

**Keywords:** bicytopenia, copper deficiency, cytopenia, cytoplasmic vacuolization, myelodysplastic syndrome, short bowel syndrome, total parenteral nutrition, trace elements

## Abstract

The patient was a 24-year-old female with pyruvate dehydrogenase complex deficiency and cerebral palsy. At 14 years of age, she developed short bowel syndrome due to intestinal obstruction and began receiving total parenteral nutrition (TPN) at home. At 15 years of age, the patient developed intestinal failure associated with liver disease. At 23 years of age, she rapidly developed jaundice and liver dysfunction. Liver biopsy revealed significant iron deposition, which led to the discontinuation of trace element supplementation.

She was referred to our department 10 months after the discontinuation of trace element supplementation because of bicytopenia (Hb 5.7 g/dL and neutrophils 1,363/μL). During the course of the illness, the neutrophil count dropped to 204/μL. Although she had macrocytic anemia (MCV 101.0 fl), no decrease in vitamin B12 or folate levels was observed. Copper was decreased to 4 μg/dL (reference range: 80-155 μg/dL), and ceruloplasmin was ≤2 mg/dL (reference range: 20-40 mg/dL). Bone marrow examination revealed hyperplastic marrow with dysplasia in the erythroid lineage and cytoplasmic vacuolation in both the erythroid and granulocytic lineages, but no increase in blasts and a normal karyotype. Anemia due to zinc deficiency typically presents as normocytic or microcytic anemia. Based on the macrocytic anemia and characteristic bone marrow findings, copper deficiency was considered the cause of the hematopoietic disorder; therefore, trace element supplementation was resumed. After red blood cell transfusion and the initiation of trace element supplementation, an increase in copper levels was confirmed; anemia improved on the fourth day and remained stable, while neutrophil counts recovered within two weeks.

This case was considered to be a result of copper deficiency due to multiple factors, including malabsorption due to short bowel syndrome, long-term TPN administration, and discontinuation of trace element supplementation, leading to hematopoietic dysfunction. Recently, deficiencies in trace elements such as zinc and selenium during parenteral nutrition have been highlighted, and attention to copper deficiency is also warranted. Regular monitoring and careful dose adjustments are essential in children at high risk of trace element deficiency. Furthermore, cytopenia caused by copper deficiency may require differentiation from myelodysplastic syndrome based on bone marrow findings. However, because hematologic recovery is typically promptly observed with copper supplementation, accurate diagnosis and treatment are crucial.

## Introduction

Copper is an essential trace metal that plays an important role in many enzymes and electron transport proteins, as well as a cofactor in antioxidant metabolism. Causes of copper deficiency include hereditary diseases such as Menkes disease, upper gastrointestinal surgery, malabsorption syndrome, and total parenteral nutrition (TPN) [1,2]. Although copper is widely distributed in the diet and copper deficiency remains rare, copper deficiency has been reported to cause hematological abnormalities such as anemia and neutropenia, which may mimic myelodysplastic syndromes (MDS) [3]. The pathogenesis involves impaired iron metabolism, oxidative damage to hematopoietic cells, and disruption of hematopoiesis [4-7].

There are a few reports of copper deficiency-induced bicytopenia in patients with short bowel syndrome receiving long-term TPN [2,8]. However, as patients can choose to receive TPN at home, and because of the aging population and long-term survival of patients with short bowel syndrome due to necrotizing enterocolitis or intestinal obstruction, it is expected that the number of patients dependent on long-term TPN use will increase. We present a case of copper deficiency resulting in bicytopenia in a patient with short bowel syndrome receiving TPN, along with a review of the literature.

## Case presentation

A 24-year-old female with short bowel syndrome who had been on TPN for 10 years presented to the emergency department with fever; she was found to have bicytopenia and was referred to our division. She was diagnosed with ventriculomegaly during the fetal period and pyruvate dehydrogenase complex (PDHC) deficiency after birth. She had cerebral palsy and profound psychomotor developmental delay, requiring full assistance with activities of daily living. She was treated for central hypogonadism using an estradiol patch. Additionally, she was under observation without medication for symptomatic epilepsy, osteoporosis (Z score = -6.0), and central hypothyroidism. She had experienced aerophagia and gastric volvulus many times; therefore, laparoscopic gastropexy was performed at the age of 13 years. At 14 years of age, the intestine was incarcerated above the site of gastropexy, leading to intestinal obstruction, for which gastrostomy and colectomy were performed. Subsequently, she developed intestinal volvulus, resulting in intestinal obstruction and extensive bowel necrosis, requiring bowel resection and high jejunocolic anastomosis, leaving only 10 cm of preserved bowel. Therefore, she was diagnosed with short bowel syndrome. Since then, she has been receiving high-calorie infusions via a central venous catheter at home, including lipid emulsions, amino acids, vitamins, selenium, carnitine, and thiamine, and enteral nutrition and blended food via gastrostomy. At the age of 15 years, she developed intestinal failure-associated liver disease. At the age of 23 years, she rapidly developed jaundice and worsened liver dysfunction, and a liver biopsy performed for further evaluation revealed significant iron deposition in the liver. Trace element supplementation, suspected to be a contributing factor to liver dysfunction, was discontinued from TPN 10 months prior to hospitalization, along with a glucagon-like peptide-2 (GLP-2) analog, which was also suspected.

After admission, she received two units of red blood cells and was referred to our department for further evaluation of cytopenia. At the time of referral, she had no fever, her vital signs were stable, and she was alert and responsive. Her face showed mild pallor, and paleness was observed in the conjunctivae. No petechiae were observed in the oral cavity; however, subcutaneous hemorrhages were noted on her extremities. Laboratory results are presented in Table 1.

**Table 1:** Laboratory evaluations Hct: Hematocrit; MCV: Mean corpuscular volume; MCHC: Mean corpuscular hemoglobin concentration; RDW: Red cell distribution width; Ret: Reticulocyte count; PLT: Platelet count; Na: Sodium; K: Potassium; Cl: Chlorine; Mg: Magnesium; Cu: Copper; Zn: Zinc; T-Bil: Total bilirubin; D-Bil: Direct bilirubin; AST: Aspartate aminotransferase; ALT: Alanine aminotransferase; LDH: Lactate dehydrogenase; γGTP: Gamma-glutamyl transpeptidase; CK: Creatine kinase; TP: Total protein; Alb: Albumin; BUN: Blood urea nitrogen; Cre: Creatinine; CRP: C-reactive protein; Fe: Iron; UIBC: Unsaturated iron binding capacity; TIBC: Total iron binding capacity.

Parameters	Actual values	Reference values
Complete blood count (CBC)		
WBC (/μL)	2,500	4,500-11,000
Neutrophil (%)	54.5	40-70
Lymphocyte (%)	13	20-45
Hemoglobin (Hb) (g/dL)	5.7	11.6-14.8
Hct (%)	17.5	35-45
MCV (fl)	101.7	83-99
MCHC (g/dL)	32.6	31-36
RDW (%)	19.2	11.5-13.8
Ret (‰)	16	5-20
Ret (×10^4/^/μL)	2.65	2.5-10
PLT (×10^4^/μL)	15	15-40
Biochemistry		
Na (mEq/L)	139	138-145
K (mEq/L)	3.9	3.6-4.8
Cl (mEq/L)	103	101-108
Ca (mg/dL)	8.6	8.7-10.3
IP (mg/dL)	3	2.5-4.5
Mg (mg/dL)	1.5	1.8-2.3
Cu (μg/dL)	4	80-155
Zn (μg/dL)	10	65-110
Ceruloplasmin (mg/dL)	≦2	20-40
T-Bil (mg/dL)	0.9	0.2-1.2
D-Bil (mg/dL)	0.4	0.0-0.3
AST (U/L)	46	13-30
ALT (U/L)	60	10-42
LDH (U/L)	156	119-229
γGTP (U/L)	307	7-23
CK (U/L)	16	41-153
TP (g/dL)	5.7	6.5-8.0
Alb (g/dL)	3.1	4.1-5.1
BUN (mg/dL)	10.5	8-20
Cre (mg/dL)	0.3	0.5-0.8
CRP (mg/dL)	0.55	0.00-0.30
Fe (μg/dL)	75	48-170
UIBC (μg/dL)	97	150-300
TIBC (μg/dL)	172	250-400
Ferritin (ng/mL)	2,614	10-200
Vitamin B_12_ (pg/mL)	21,100	180-914
Vitamin C (μg/mL)	5.6	6-20
Folic acid (ng/mL)	17.2	4.0-20.0
Antinuclear antibodies (×)	<40	<40
Bone marrow examination		
Cell count (/μL)	278,000	50,000-100,000
Megakaryocyte count (/μL)	219	50-150
Granulocyte series (%)	25.8	50-70
Erythrocyte series (%)	63.4	20-30
Lymphocyte series (%)	7	10-20
Blast (%)	0	<1.0
G-band	46, XX	
-7/del(7q) (%)	0.0	〜2-3
+8 (%)	0.0	〜2-3

Complete blood count (CBC) revealed macrocytic normochromic anemia, low neutrophil count, mild thrombocytopenia, and a decrease in reticulocyte count, and she developed neutropenia during her hospital stay. No decrease in vitamin B12 or folate levels was observed. Prothrombin time-International normalized ratio (PT-INR) and activated partial thromboplastn time (APTT) were prolonged, and D-dimer levels were mildly elevated. Electrolyte levels were normal, and liver transaminase levels were elevated, similar to her baseline levels. Although she was underweight, with a height of 134 cm (−4.5 SD) and a weight of 21 kg (−4.1 SD), her nutritional status was not impaired, with her total protein level of 5.7 g/dL and serum albumin of 3.1 g/dL, both within the normal range. There was no evidence of iron deficiency.

**Figure 1:**
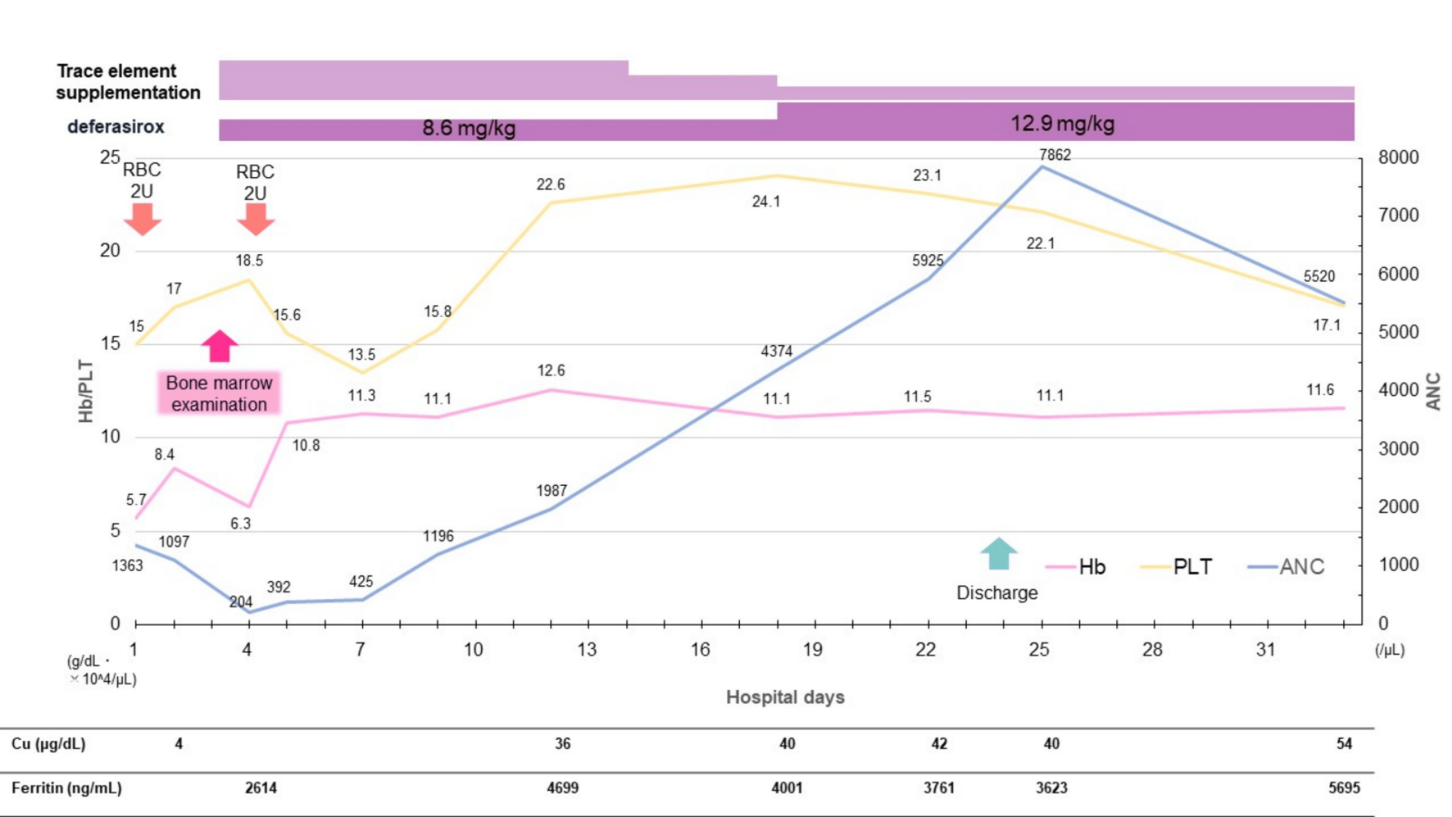
Progress after hospitalization On day 3 of hospitalization, bone marrow examination revealed vacuolization and erythroid dysplasia. Serum copper was markedly decreased to 4 μg/dL, and ceruloplasmin was ≤2 mg/dL, confirming copper deficiency. Trace element supplementation was initiated, and anemia stabilized by day 4. Neutrophil count recovered within two weeks, and the patient was discharged on day 24 after adjustment of trace element therapy.

Progress after hospitalization is shown in Figure 1. On the third day of hospitalization, a bone marrow examination revealed hypercellular marrow with hyperplasia and mild dysplasia in the erythroid lineage.

**Figure 2:**
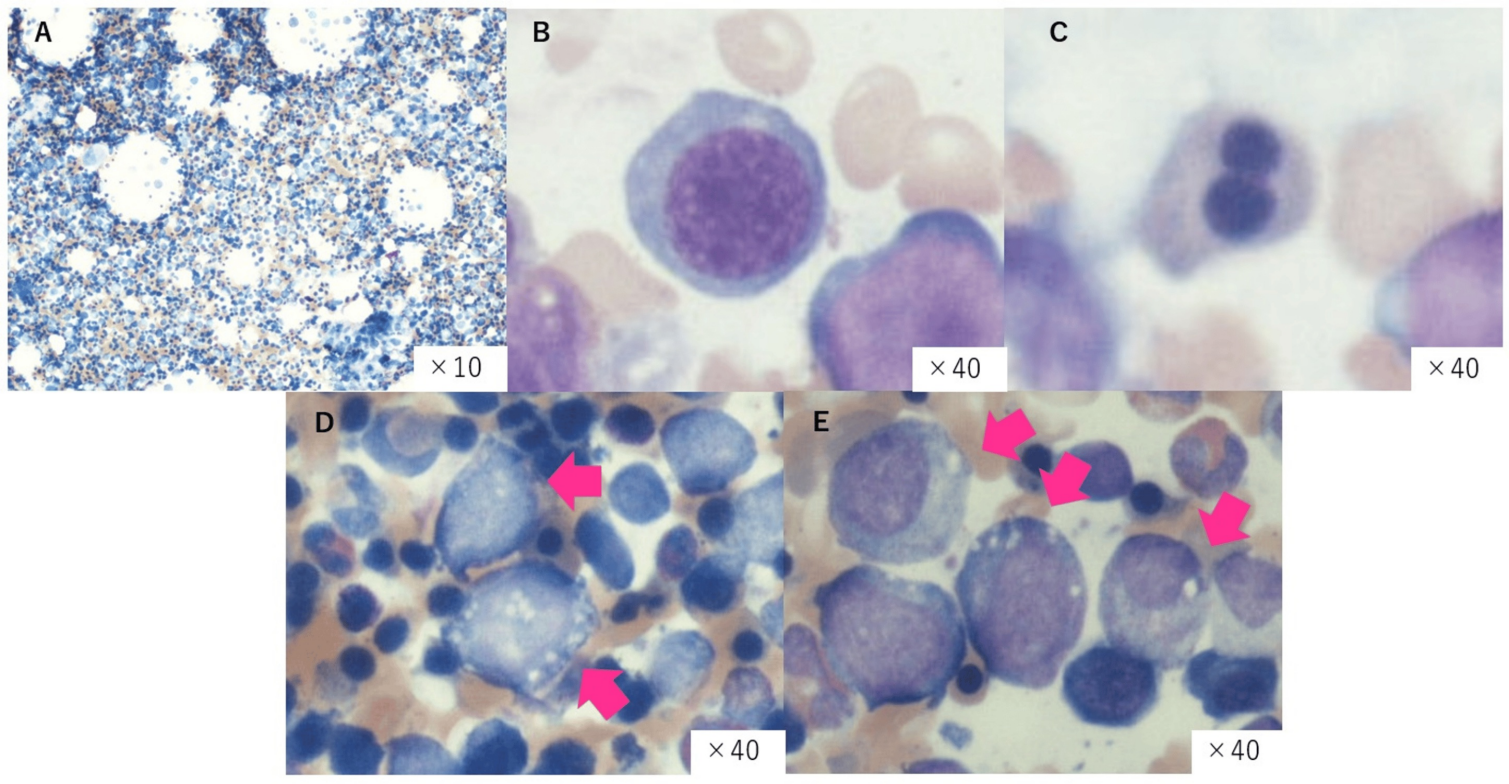
Bone marrow findings The macroscopic findings of the bone marrow examination (×10) revealed hyperplastic bone marrow, with marked hyperplasia of the erythroid lineage (A). In the magnified view, megaloblastic changes (B), binucleated erythroblasts (C), and cytoplasmic vacuolation in erythroid and granulocytic lineages (D,E) were observed.

Vacuolization was also observed in the cytoplasm of the erythroid and granulocytic lineages (Figures 2A-2E). There was no increase in the number of blasts or numerical chromosomal abnormalities. On the same day, it was confirmed that the copper level was decreased to 4 μg/dL (reference range: 80-155 μg/dL), and the ceruloplasmin level was ≤2 mg/dL (reference range: 20-40 mg/dL). Based on the macrocytic anemia and characteristic bone marrow findings, copper deficiency was identified as the cause of the hematopoietic disorder, and trace element supplementation was resumed. Subsequently, marked cardiomegaly and pleural effusion developed, and as the edema worsened, furosemide was started and its dosage was adjusted. After copper supplementation, anemia stabilized by the fourth day, neutrophil levels recovered within two weeks, and both heart failure and pleural effusion improved. The furosemide dose was gradually tapered, and there was no recurrence of heart failure. After adjusting trace element dosages, the patient was discharged on the 24th day of hospitalization.

The currently available trace element preparation is a combined formulation of iron, copper, zinc, manganese, and iodine. Since discharge, the dosage of the trace element preparation has been carefully adjusted to avoid exacerbation of iron overload, while serum copper levels are being monitored. Cocoa was used concurrently as a source of copper supplementation, and serum copper levels were maintained at approximately 50 μg/dL. However, because of significant diarrhea, which is considered a side effect of malabsorption in short bowel syndrome, cocoa was discontinued. As of eight months after discharge, copper levels have remained within the range of 24-57 μg/dL, with no evidence of cytopenia observed during follow-up.

## Discussion

Copper is the third most abundant trace element in the human body, after iron and zinc. It is widely distributed in the diet, and the daily requirement for copper varies across all ages: 20 μg/kg/day for children aged less than five years, 0.3 to 0.5 mg/day for children aged more than five years, and 2-3 mg/day for an adult [1]. The primary locations of copper absorption are the duodenum and, to a lesser extent, the stomach. The causes of copper deficiency vary and include genetic disorders such as Menkes disease, and malabsorption due to conditions such as upper gastrointestinal surgeries, short bowel syndrome, or celiac disease [1,2]. Other causes include excessive zinc intake, long-term TPN, increased demand during pregnancy, and drug-induced factors [1,2]. A prospective study of children with intestinal failure receiving home TPN identified several factors associated with lower serum copper levels, including the duration of TPN without copper supplementation, elevated direct bilirubin levels, and having an ostomy (jejunostomy and ileostomy) [9]. In this case, 16 months before admission, the patient experienced a sudden onset of jaundice and liver dysfunction, and a liver biopsy revealed significant iron deposition. As a result, trace element supplements containing iron, copper, and zinc simultaneously, along with the suspected medication, were discontinued 10 months before admission, and the liver dysfunction gradually improved over time. Although the cause of jaundice was unclear, iron overload due to iron in the TPN or side effects of the GLP-2 agonist were considered. We propose that the causes of copper deficiency in this case were multifactorial and included impaired absorption due to short bowel syndrome, extended dependence on TPN, and cessation of copper supplementation for 10 months.

The association between copper deficiency and cytopenia in humans was first identified by Cordano in 1966, who observed a strong correlation between absolute neutrophil count and serum copper levels in malnourished children from Peru [10]. However, the underlying mechanisms behind this association remain unclear. Several hypotheses have been proposed. In terms of anemia, (1) the reduction of ceruloplasmin leads to impaired conversion of Fe^2+^ to Fe^3+^, which hinders iron transport by transferrin, resulting in defective iron utilization [4]; (2) decreased activity of the copper-dependent enzyme superoxide dismutase reduces the removal of free radicals, leading to damage to the cell membrane and shortened lifespan of red blood cells [5]; and (3) decreased activity of the copper-containing enzyme cytochrome c oxidase impairs iron reduction, which disrupts heme synthesis [6]. The etiology of neutropenia is less well understood than that of anemia. The following have been suggested [5,11]: (1) destruction and maturation arrest of bone marrow precursor cells, (2) impaired migration of neutrophils from the bone marrow, and (3) increased clearance of neutrophils from the circulation.

In this case, although some morphological abnormalities were observed in the erythroid lineage on bone marrow examination, the characteristic findings of copper deficiency, such as cytoplasmic vacuolation in both the erythroid and granulocyte lineages, were prominent. Additionally, none of the typical findings associated with MDS, including chromosomal abnormalities, pseudo-Pelger-Huët anomalies, or dysplastic features in the myeloid or megakaryocytic lineages, were detected. While the patient also had hypozincemia, and the trace element preparation contained zinc, making it difficult to completely rule out the influence of zinc deficiency on cytopenia, anemia due to zinc deficiency typically presents as microcytic to normocytic anemia and does not lead to neutropenia. Given the macrocytic anemia and characteristic bone marrow findings, copper deficiency was considered the primary cause of the hematopoietic disorder. The patient’s cytopenia rapidly improved after supplementation with trace elements.

When copper deficiency leads to hematological abnormalities, it commonly presents with neutropenia and anemia, which involve reductions in the two blood cell lines. However, thrombocytopenia is rarely observed. Halfdanarson et al. reviewed the hematological findings in 40 adults with copper deficiency and reported that 52.5%, 30%, 10%, 5%, and 2.5% of patients had anemia and leukopenia, isolated anemia, pancytopenia, anemia and thrombocytopenia, and isolated neutropenia, respectively [7]. Additionally, there was a correlation between low serum copper levels and low white blood cell (r = 0.37, p = 0.025) and neutrophil counts (r = 0.47, p = 0.003). Haddad et al. summarized the hematological findings of 48 patients with copper deficiency from the literature, showing that anemia was present in 100% and neutropenia in 98% of the patients, but thrombocytopenia was only observed in 10% [12].

According to the study by Halfdanarson et al. [7], bone marrow findings in copper deficiency showed vacuolization of proerythroblasts and myelocytes in 88% of cases and relative erythroid hyperplasia in 81% of cases. Additionally, silver-positive staining was observed in the macrophages (69%) and plasma cells (56%), with ringed sideroblasts occasionally present (31%). These findings, along with dysplasia, require differentiation from those of MDS. In fact, it has been reported that seven of eight patients diagnosed with cytopenia due to copper deficiency had been previously diagnosed with MDS based on bone marrow examinations conducted at previous medical facilities, and three of these patients were referred for consideration of allogeneic stem cell transplantation [3]. Certain hematological findings in copper deficiency resemble those observed in MDS. In copper deficiency, findings that aid in distinguishing it from MDS include the absence of abnormalities commonly associated with MDS, such as pseudo-Pelger-Huët anomaly, hypogranular neutrophils, nuclear fragmentation in erythroblasts, and megakaryocytic abnormalities. Instead, cytoplasmic vacuolization of erythroid and myeloid precursor cells is a characteristic feature [3,13]. Cytopenia caused by copper deficiency improves promptly with copper supplementation. Given that the treatment differs significantly from that for MDS, an accurate diagnosis is essential.

There are a few reports of cytopenia involving two or more cell lineages due to copper deficiency associated with short bowel syndrome. To the best of our knowledge, this is the ninth reported case to date. A summary of previous cases is presented in Table 2 [2,8,14-18].

**Table 2:** Previous Cases of Copper Deficiency with Cytopenia of Two or More Blood Systems in Patients with Short Bowel Syndrome TPN: Total parenteral nutrition; PPN: Partial parenteral nutrition; G-tube: Gastrostomy tube; MR: Mitral regurgitation; AR: Aortic valve insufficiency; Cytopenia: Hb ≦11 g/dL; Neutropenia: Neut≦1,000/μL; Thrombocytopenia, ◯: ≦10×10^4^/μL, △: 10×10^4^＜ ≦15×10^4^/μL.

No.	reference	Age	Sex	Cytopenia			Cu(μg/dL)	vacuolization	Years since developing short bowel syndrome	Length of preserved intestine	Nutritional pathway	Cholestasis	Other associated symptoms
				Anemia	Neutropenia	Thrombocytopenia							
1	Angotti et al., 2008 [14]	9 m	M	◯	◯	◯	2	Yes	about one month	15 cm	TPN	Yes	Hepatosplenomegaly
2	Yu et al., 2019 [15]	5 y	F	◯	◯	×	ー	Yes	ー	ー	TPN	No	
3	Dembinski et al., 2012 [8]	6 y	M	◯	×	△	84	ー	ー	ー	TPN + G-tube	Yes	
														4	Dembinski et al., 2012 [8]	12 y	M	◯	◯	◯	4	No	ー	ー	TPN + G-tube	No	
5	Spiegel et al., 1999 [16]	32 y	M	◯	◯	△	17	Yes	ー	100 cm	TPN + G-tube	Yes	Sepsis and uremia. The patient died from cardiac tamponade two weeks after the resolution of cytopenia following copper supplementation.														
6	Fuhrman et al., 2000 [17]	36 y	F	◯	◯	◯	25	Yes	ー	ー	TPN	No	The patient, who had complications of heart failure due to MR, AR, liver fibrosis, fatty liver, ascites, hepatic encephalopathy, intra-abdominal bleeding, and splenomegaly, passed away four months after copper supplementation.
7	Hantaweepant et al., 2016 [2]	37 y	M	◯	◯	×	0.2	Yes	13 years	ー	PPN	No	-
8	Miki et al., 2007 [18]	48 y	M	◯	◯	△	3	Yes	35 years	ー	TPN	No	Bradycardia, gait disturbance, and limb weakness.
9	Present case	24 y	F	◯	◯	△	4	Yes	10 years	12 cm	TPN + G-tube	Yes	Heart failure, osteoporosis

Cytopenia was defined as hemoglobin (Hb) ≤ 11 g/dL, neutrophils ≤ 1,000/μL, and platelets ≤ 10×10^4^/μL, with reduction of platelet counts to 10×10^4^ to 15×10^4^/μL marked as △. Anemia was observed in all previous cases, and neutropenia was noted in all but one case. In contrast, thrombocytopenia was less frequent, being observed in only 33% of cases; however, when mild thrombocytopenia was included, this figure increased to 78%. While details were unclear in many cases, all patients received TPN, and cholestasis occurred in five out of nine cases. Although no literature was found that generally describes the time interval from copper removal to the development of cytopenia, the references listed in Table 2 report durations of five months [14], eight months [16], and 15 months [17]. In the present case, the interval was 10 months. In other words, in patients with impaired copper absorption who rely on TPN for trace elements, cytopenia may develop approximately six months to one year after copper removal. In this case, discontinuation of the iron-containing trace element preparation was necessary to prevent the progression of hepatic dysfunction caused by iron overload, which subsequently led to copper deficiency. Copper deficiency is treated with oral or intravenous copper replacement in the form of copper gluconate, copper sulfate, or copper chloride [19]. Although the dose of the trace element preparation is carefully adjusted while monitoring ferritin and serum copper levels with the concurrent use of an iron chelator, iron overload, which initially led to the discontinuation of the trace element preparation, persists. Although cytopenia due to copper deficiency has not occurred, the underlying cause remains unresolved. Several reports from Japan have described the use of cocoa as an oral source of copper supplementation [20], and in the present case, cocoa was also administered via gastrostomy after discharge; however, because of malabsorption associated with ultrashort bowel syndrome, severe diarrhea developed, making continued administration difficult. With the increasing long-term survival of patients with short bowel syndrome and the aging population, cases involving trace element deficiencies and the dilemmas associated with supplementation therapy, such as in this case, are expected to become more common in the future.

One limitation of this case is the presence of an underlying condition, PDHC deficiency, making it difficult to determine the extent to which it contributed to the anemia. However, hematologic parameters improved following trace element supplementation, suggesting that the impact of the underlying condition was likely minimal.

## Conclusions

We present a case of bicytopenia caused by copper deficiency in a patient with short bowel syndrome receiving long-term TPN. While cytopenia due to copper deficiency is rare, recent prospective studies have been conducted, and cases are gradually accumulating. There is a possibility of misdiagnosis of MDS based on hematological and bone marrow findings; however, an accurate diagnosis is crucial, as rapid improvement can be achieved with copper supplementation. We encourage physicians to consider copper deficiency when cytopenia and morphological abnormalities of the bone marrow, particularly cytoplasmic vacuolization, are not accompanied by cytogenetic abnormalities, especially in patients with a history of gastrointestinal surgery, malabsorption syndromes, or long-term TPN. Therefore, it is necessary to monitor copper levels regularly and carefully adjust supplementation based on serum values and clinical symptoms.

In this case, the elevated ferritin levels that initially led to the discontinuation of trace element supplementation have persisted despite the use of deferasirox. We carefully adjust the trace element supplementation while balancing serum copper levels. This case highlights the clinical dilemma of managing trace element deficiencies and supplementation, particularly the complexity of maintaining appropriate levels of both iron and copper.
